# Stress induces microglia-associated synaptic circuit alterations in the dorsomedial prefrontal cortex

**DOI:** 10.1016/j.ynstr.2021.100342

**Published:** 2021-05-20

**Authors:** Taohui Liu, Ju Lu, Kacper Lukasiewicz, Bingxing Pan, Yi Zuo

**Affiliations:** a School of Life Science, Nanchang University, Nanchang, Jiangxi, 330031, China; b Department of Molecular, Cell and Developmental Biology, University of California Santa Cruz, 1156 High Street, Santa Cruz, CA, 95064, USA

**Keywords:** Stress, Dendritic spine, Microglia, Cognitive flexibility, Prefrontal cortex

## Abstract

The mammalian dorsomedial prefrontal cortex (dmPFC) receives diverse inputs and plays important roles in adaptive behavior and cognitive flexibility. Stress, a major risk factor for many psychiatric disorders, compromises the structure and function of multiple brain regions and circuits. Here we show that 7-day restraint stress impairs reversal learning in the 4-choice odor discrimination test, a decision-making task requiring an intact dmPFC. *In vivo* two-photon imaging further reveals that stress increases dmPFC dendritic spine elimination, particularly those of the mushroom morphology, without affecting spine formation. In addition, stress alters dmPFC microglial branching complexity and elevates their terminal process dynamics. In stressed mice, dmPFC microglia contact dendrites more frequently, and dendritic spines with microglial contact are prone to elimination. In summary, our work suggests that stress-induced changes in glial-synapse interaction contributes to synaptic loss in dmPFC, resulting in neuronal circuit deficits and impaired cognitive flexibility.

## Highlights

• Restraint stress impairs cognitive flexibility in adolescent mice.
• Stress leads to synapse loss on pyramidal neurons in the dorsomedial prefrontal cortex.
• Stress decreases microglial complexity but increases their terminal dynamics and contacts with dendritic spines.
• Dendritic spines contacted by microglial processes are more prone to elimination.

## 1 Introduction

Stress is common in modern life. With deleterious impacts ranging from increased anxiety level to cognitive impairment, stress is a major risk factor for many psychiatric disorders, including schizophrenia, generalized anxiety disorder, major depressive disorder, bipolar disorder, and post-traumatic stress disorder (Chrousos, 2009; de Kloet et al., 2005). Stress compromises the structure and function of multiple brain regions and circuits (Gold, 2015; McEwen, 2007; McEwen et al., 2016). The prefrontal cortex (PFC), a brain region affected in many psychiatric disorders, is one of the main neuropathological targets of stress (Arnsten, 2009, 2015). PFC connects with many cortical and subcortical regions and contributes to diverse cognitive functions (Euston et al., 2012; Matyas et al., 2014; Varela et al., 2014). Compared to sensorimotor cortices, PFC development is protracted, with its maturation continuing into the third decade of life in humans (Kolb et al., 2012). Thus, disturbances to the PFC during adolescence may underlie the susceptibility to neuropsychiatric disorders (Caballero and Tseng, 2016; Casey et al., 2008; Gamo and Arnsten, 2011; Paus et al., 2008). The rodent PFC has several sub-regions with distinct connectivity and functions (Barbas, 2015; Barbas and Zikopoulos, 2007). Among them, the dorsomedial PFC (dmPFC) plays a critical role in the flexible control of voluntary actions and adaptive action selection (Barthas and Kwan, 2017; Ebbesen et al., 2018). Activities of dmPFC neurons can convey information about past choice and outcome (Siniscalchi et al., 2019; Sul et al., 2011), and dmPFC removal or inactivation impairs cue-guided actions (Johnson and Wilbrecht, 2011; Wang et al., 2020). However, we have limited knowledge about how stress affects dmPFC despite its prominence in higher cognitive functions.

Rodent studies have revealed that stress induces significant changes in dendritic morphology and dendritic spines of neurons in the PFC, hippocampus, and amygdala (Christoffel et al., 2011; McEwen et al., 2016; Radley and Morrison, 2005). Such morphological changes vary among brain regions and stress types. For example, chronic restraint stress causes dendritic retraction and decreases spine density in the anterior cingulate cortex and the prelimbic area of PFC (Hains et al., 2009; Liu and Aghajanian, 2008; Radley et al., 2006, 2008). These stress-induced alterations are associated with deficits in executive functions such as working memory and cognitive flexibility (Liston et al., 2006; Radley et al., 2015), as well as emotional dysregulation as evidenced by impaired fear extinction (Holmes and Wellman, 2009). Chronic stress likewise induces dendritic atrophy and spine loss of pyramidal neurons in hippocampal CA1 and CA3 regions (Watanabe et al., 1992), impairing long-term potentiation and leading to memory deficits (Kim et al., 2015; Sousa et al., 2000). In contrast, chronic stress increases spine density on pyramidal and stellate neurons in the basolateral amygdala, increasing anxiety and aggression (Vyas et al., 2002; Wood et al., 2003). It also increases dendritic branching of pyramidal neurons in the orbitofrontal cortex (Liston et al., 2006). Collectively, these works indicate that the impact of stress is circuit-specific and regimen-dependent.

Microglia are the resident immune cells in the brain, carrying out a variety of functions therein (Bachiller et al., 2018; Prinz et al., 2019; Salter and Stevens, 2017; Wolf et al., 2017). In the healthy brain, quiescent microglia continuously extend and retract their processes (Davalos et al., 2005; Nimmerjahn et al., 2005) and contact neuronal structures (Miyamoto et al., 2016; Tremblay, 2011; Wake et al., 2009). It is increasingly recognized that a delicate reciprocal interaction exists between microglia and neurons. On the one hand, neuronal network activity regulates microglial process dynamics and surveillance (Eyo et al., 2015; Liu et al., 2019); on the other hand, microglia modulate synaptic pruning and plasticity, shape synaptic transmission, and ultimately contribute to behavioral regulations (Hoshiko et al., 2012; Miyamoto et al., 2016; Paolicelli et al., 2011; Parkhurst et al., 2013; Rogers et al., 2011; Schafer et al., 2012; Sipe et al., 2016; Tremblay et al., 2010; Wang et al., 2020; Xavier et al., 2014). Stress induces changes in microglial morphology and reactivity (Calcia et al., 2016; Frank et al., 2019; Tynan et al., 2010; Walker et al., 2014). In stress-sensitive brain areas such as the prelimbic, the infralimbic, and the anterior cingulate regions of PFC, chronic stress causes microglia activation and increases the branch complexity of microglia processes (Hinwood et al., 2012; Tynan et al., 2010). Such microglial hyper-ramification coincides with alterations in neuronal activity and working memory (Hinwood et al., 2012, 2013; Kopp et al., 2013). However, it remains unclear how microglia changes are linked to synaptic alterations in response to stress.

## 2 Materials and methods

### 2.1 Animals

The *thy1*-YFP-H (JAX#003782) and Cx3cr1-GFP (JAX#005582) mouse lines were purchased from The Jackson Laboratory. All mice were backcrossed into the C57BL/6J background. The two lines were crossed to generate YFP+/GFP+ mice for co-imaging of dendritic spines and microglia. Mice were group-housed in the UCSC animal facility, with 12 h light-dark cycle and access to food and water *ad libitum*. Both sexes were used in all experiments. All animal studies were performed in accordance with the National Institutes of Health guide for the care and use of laboratory animals, following protocols approved by the Animal Care and Use Committee (IACUC) of UCSC.

### 2.2 Restraint stress

We followed the protocol described previously (Chen et al., 2018). Briefly, the mouse was placed into a perforated 50 ml conical tube for 2 h daily for 7 consecutive days, starting at one month of age.

### 2.3 Four-choice odor discrimination and reversal test

We followed the protocol described previously (Johnson and Wilbrecht, 2011) with slight modifications. Briefly, the mouse was trained to explore a custom-made 4-chamber arena and dig for a food reward (~10 mg piece of Honey Nut Cheerio) covered by wood shavings in ramekins associated with one of the four odors. Testing consisted of a discrimination session followed by a reversal session. During the discrimination session, the mouse discriminated among four odors (rosemary, clove, thyme, and nutmeg) and learned that the reward was associated with rosemary. The session criterion was met if the mouse correctly completed 8 out of 10 consecutive trials. During the reversal session, thyme was replaced by a novel odor (cinnamon), and food reward was associated with clove instead. Digging in the ramekin with the previously rewarded odor (rosemary) was recorded as “Perseverative Error”; digging in the ramekin with the previously presented odor that was never rewarded (nutmeg) was recorded as “Irrelevant Error”; digging in the ramekin with the newly introduced odor (cinnamon) was recorded as “Novel Error”. The same session criterion applied.

### 2.4 Y-maze spontaneous alternation test

We custom-made an opaque plastic Y-maze composed of three 35 cm × 7 cm arms diverging at 120° angles. During the test, the subject mouse was placed at the center of the Y-maze and allowed to roam freely for 15 min. Mouse behavior was recorded using an ELP USB camera with 2.8–12 mm VARIFOCAL lens (Cat# ELP-USBFHD04H-FV) and analyzed with a custom-written program in Bonsai (Lopes et al., 2015). Mouse body position was tracked in recorded videos using DeepLabCut (Mathis et al., 2018). Arm entry was counted when all limbs of the mouse were within the arm. Alternation was defined as a series of three consecutive arm entries into three unique arms. Percent alternation was calculated by dividing the number of observed alternations by the maximum number of alternations multiplied by 100%.

### 2.5 Immunohistochemistry of microglia density and neuronal c-Fos expression

The mouse was transcardially perfused as previously described (Hodges et al., 2017). The brain was cut into 40 μm-thick coronal sections with a vibratome (VT1000S, Leica Biosystems Inc.).

To examine microglia density, brain sections were incubated in a solution of 10% (w/v) normal rabbit serum, 5% BSA, and 0.7% PBST for 2 h to block non-specific binding, followed by incubation with a goat anti-Iba1 primary antibody (ab5076, Abcam, 1:1000) at 4 °C for 72 h. Sections were then incubated with a rabbit anti-goat secondary antibody conjugated to AlexaFluor 594 (A11080, ThermoFisher Scientific; 1:500) for 2 h at room temperature, rinsed in PBS, and mounted on slides with the mounting medium Fluoromount-G (0100–01, SouthernBiotech). Images were captured on a Zeiss AxioImager Z2 widefield microscope using a 10x/0.45 NA air objective. Microglia density (number of microglia per mm^2^) was quantified using Neurolucida Explorer 11 (MBF Bioscience).

To examine the neuronal expression of c-Fos, the mouse was perfused 1 h after the end of the last session of 7d RS or the end of the 4-choice task. Brain sections were incubated in a solution of 10% (w/v) normal goat serum, 5% BSA, and 0.7% PBST for 2 h to block non-specific binding, followed by incubation with a rabbit anti-c-Fos primary antibody (ab7963, Abcam; 1:1000) at 4 °C for 72 h. Sections were then incubated with a goat anti-rabbit secondary antibody conjugated to AlexaFluor 488 (A11008, ThermoFisher Scientific; 1:500) for 2 h at room temperature, rinsed in PBS, and mounted on slides with the mounting medium Fluoromount-G. Image acquisition and quantification of the density of c-Fos + neurons were performed as above.

### 2.6 Virus injection and pharmacogenetic manipulation

AAV8-CaMKlla-hM4D-mCherry (DREADD virus) and AAV2-CaMKlla-mCherry (control virus) were purchased from the University of North Carolina Vector Core. For virus injection, P21 mice were anesthetized with isoflurane (4% for induction, 1.5% for maintenance). Dexamethasone (2 mg/kg bodyweight) was injected intramuscularly, and carprofen (5 mg/kg bodyweight) was injected intraperitoneally (i.p.). 200 nl virus was injected into dmPFC (AP +1.7 mm, ML 0.6 mm, depth −1.32 mm) at 40 nl/min using a custom-built injection system based on a single-axis oil hydraulic micromanipulator (MO-10, Narishige). The mouse received the analgesic buprenorphine (0.1 mg/kg, subcutaneous) postoperatively for 3 days. 2–3 weeks of incubation was allowed before the commencement of behavioral experiments. For pharmacogenetic manipulation, clozapine-N-oxide (CNO; 0.3 mg/kg body weight) was dissolved in sterile saline and injected i.p. 15 min prior to behavioral experiments. For vehicle control, saline was injected at the same time point instead. Viral injection sites were verified by *post mortem* examination of the co-expressed mCherry in brain slices.

### 2.7 In vivo two-photon (2P) imaging of dendritic spine and microglia

Transcranial 2P imaging was performed with the thin-skull preparation as described previously (Xu et al., 2009; Yu and Zuo, 2014). Briefly, the mouse was anesthetized by i.p. injection of a cocktail of ketamine (87 mg/kg bodyweight) and xylazine (8.7 mg/kg bodyweight). The skull was exposed and thinned over dmPFC (AP +1.7 mm, ML 0.7 mm). Images were taken with a 2P microscope (Ultima Investigator, Bruker Co.) equipped with a 40x/0.8 NA water-immersion objective (Olympus) and an ultra-fast 2P laser (Mai Tai HP, Spectra-Physics).

Dendritic spine dynamics were imaged with 920 nm excitation. Image stacks were taken at 3x digital zoom with 0.7 μm step size and analyzed using ImageJ. Percentage of spines formed or eliminated was calculated as the number of spines formed or eliminated divided by the number of spines counted in the first-time images. Spines were classified into four categories (mushroom, stubby, thin, and others) based on their lengths and head diameters as previously described (Zuo et al., 2005a,b).

For microglial morphology examination, image stacks were taken as above (step size = 1 μm). Microglia with all processes included in the stack were reconstructed using the Imaris software (Bitplane). Microglia processes were traced using the Imaris filament tracing function. The number of terminal points, branch numbers, and number of Sholl intersections were exported for comparison. To follow the structural dynamics of microglia, image stacks (4x zoom, 30–40 optical sections, step size = 1 μm) were taken every 5 min for 30 min total. The terminal branches of microglial processes (*i.e.*, terminal segments from the last branching point) were traced in the Imaris software; their length changes were measured over the 5 min intervals. Terminal branches that formed *de novo* after the first imaging session were excluded. We define terminal dynamics as the average absolute change in the length of the terminal at every 5-min interval within 30 min. Mathematically, let *L*_*n*_(*t*) denote the length of the *n*-th terminal of a microglia at the *t*-th imaging timepoint, the terminal dynamics of the microglia is given by $D=\frac{1}{NT}\overset{N}{\underset{n=1}{\sum }}\overset{T-1}{\underset{t=0}{\sum }}\left|L_{n}\left(t+1\right)-L_{n}\left(t\right)\right|$, where *N* is the total number of analyzed terminals of the microglia, and *T* = 6 is the total number of imaging intervals.

To co-image dendritic spines and microglia, we used a 16x/0.8 NA water-immersion objective (CFI75 LWD 16X W, Nikon Instruments, Inc.) and co-excited YFP and GFP at 940 nm. We split the emitted fluorescence with a dichroic (T565LPXR, Chroma Technology Corp.) and detected it with two photomultiplier tubes (channel 1 emission filter: ET595/50m; channel 2 emission filter: ET525/70m). Due to the spectral overlap of GFP and YFP, the shorter wavelength channel (Ch2) contained signals from both fluorophores, while the signals in the longer wavelength channel (Ch1) were predominantly from YFP. Nevertheless, neuronal and microglial structures can be unequivocally distinguished by visual inspection due to their distinct morphology, as demonstrated previously (Tremblay et al., 2010). In all subsequent analyses and illustrations, we manually segmented the contour of microglia in Ch2 and used the mask to remove the dendritic structures from the channel. We then pseudo-colored Ch1 as cyan and Ch2 as magenta. To quantify microglial contacts with dendritic shafts, we traced the dendritic segments in the cyan channel through the Z-stack and measured segmental length with overlapping cyan and magenta signals. To quantify microglial contact with dendritic spines, we identified spines in the cyan channel, and counted the number of spines with overlapping magenta signals in at least two Z-sections, similar to previously published method (Miyamoto et al., 2016).

### 2.8 Statistical analysis

Statistical tests were performed using GraphPad Prism 9.0 (GraphPad Software). The Kolmogorov-Smirnov test was used to test for sample normality to determine whether parametric or non-parametric tests were to be used. Details of statistical tests are given in the Results section. Unless stated otherwise, data are presented as mean ± s.e.m.

## 3 Results

### 3.1 7-day restraint stress impairs cognitive flexibility

**Fig. 1:**
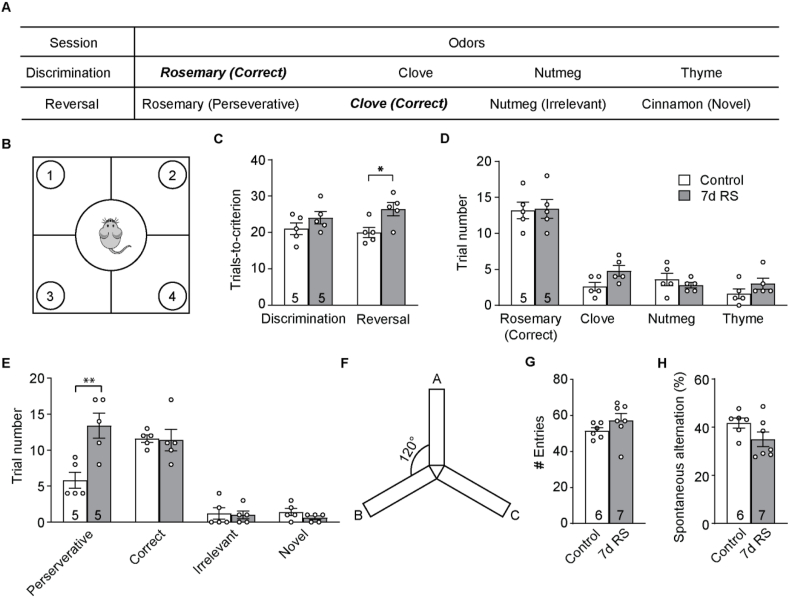
7d RS impairs cognitive flexibility of mice. (A) Odors used for the 4-choice odor discrimination and reversal task. (B) Schematic of the 4-choice test set-up. (C) Number of trials taken to reach the performance criterion in the discrimination and the reversal session. (D) Number of trials digging at each odor during the discrimination session. (E) Number of trials digging at each odor during the reversal session. (F) Schematic of the Y-maze arena. (G) Number of arm entries in control and RS mice. (H) Percentage of spontaneous alternations in control and RS mice. *p < 0.05, **p < 0.01. *n* = number of mice.

Cognitive flexibility is vital to the animal. It confers the ability to adjust the animal's behavioral strategies in response to an ever-changing environment. The four-choice odor discrimination and reversal test (“4-choice test”; Fig. 1A and B) assesses the rodent's ability to learn an odor-reward contingency and then to reverse the association (Johnson and Wilbrecht, 2011; Johnson et al., 2016). We found that adolescent mice (4–6 weeks old) passed the initial discrimination phase of the 4-choice test (performance criterion: 8 correct choices out of 10 consecutive trials) in 21.0 ± 1.6 trials. They took a comparable number of trials (20.0 ± 1.4; *p* = 0.7149, paired *t*-test; Fig. 1C) to pass the reversal phase, in which the reward was associated with a previously encountered non-rewarded odor. Among the erroneous trials, 5.8 ± 1.1 (70.4 ± 10.2%) were perseverative errors, 1.2 ± 0.8 (13.7 ± 8.6%) were irrelevant errors, and 1.4 ± 0.5 (15.9 ± 4.3%) were novel errors.

To determine how stress affects cognitive flexibility, we subjected adolescent mice to restraint stress (RS) 2 h daily for 7 days (d). One day after the last RS session they underwent the 4-choice test. We found that RS mice took 24.0 ± 1.7 trials to pass the discrimination phase, comparable to that of control mice (*p* = 0.2405, unpaired *t*-test; Fig. 1C). RS did not alter the number of digging at each odor either (Fig. 1D; *p* = 0.9215, 0.1979, 0.5704, and 0.4262 for rosemary, clove, nutmeg, and thyme, respectively, unpaired *t*-tests with FDR correction). However, they took significantly more trials than controls to pass the reversal phase (26.4 ± 1.8, *p* < 0.05, unpaired *t*-test; Fig. 1C). Interestingly, RS mice made a comparable number of correct choices as controls (*p* = 0.9119), but significantly more perseverative errors (*p* < 0.05); they committed a comparable number of irrelevant errors (*p* = 0.9119) and novel errors (*p* = 0.3939) as controls (unpaired *t*-tests with FDR correction for all four comparisons; Fig. 1E). We further tested whether the deteriorated performance might arise from a working memory deficit using the Y-maze spontaneous alternation test (Fig. 1F). We found that RS did not affect arm entries (*p* = 0.2217, unpaired *t*-test; Fig. 1G) or alternations (*p* = 0.1043, unpaired *t*-test; Fig. 1H), suggesting an intact working memory in RS mice.

### 3.2 The dmPFC is indispensable for cognitive flexibility

**Fig. 2:**
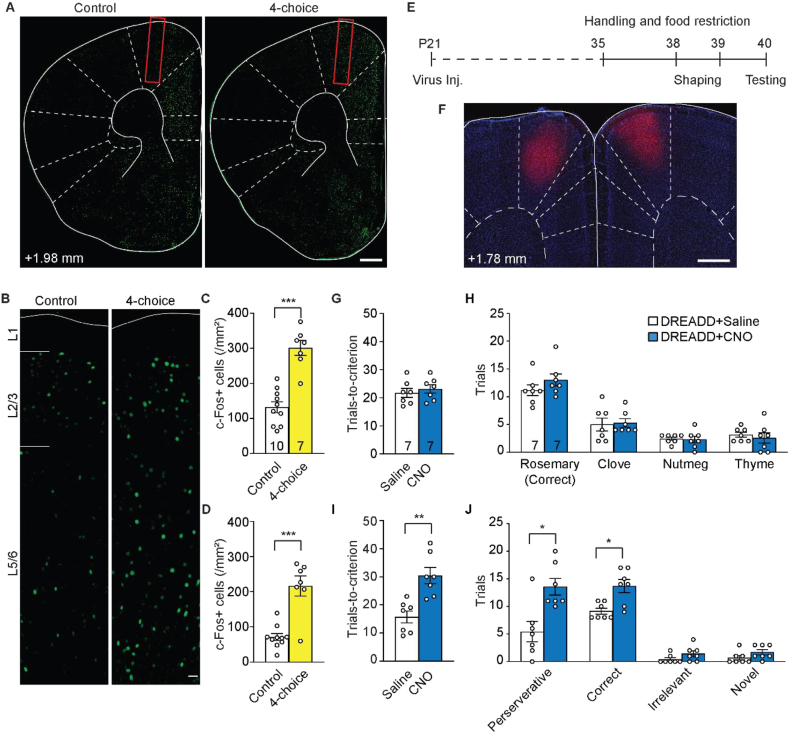
Silencing dmPFC increases perseverative errors in the 4-choice test. (A) Immunohistochemistry of c-Fos in the frontal cortex. (B) c-Fos labeling across layers (L) in dmPFC. Red rectangles: the dmPFC region analyzed. (C, D) Density of c-Fos + neurons in L2/3 (C) and L5/6 (D) dmPFC of mice with and without performing the 4-choice task. (E) Timeline of pharmacogenetic manipulation and behavioral task. (F) An example coronal section showing bilateral DREADD virus labeling in dmPFC. On average virus labeling spreads ±0.4 mm around the injection site along the anterior-posterior axis, and ±0.3 mm medial-laterally. (G) Number of trials taken to reach the performance criterion in the discrimination session. (H) Number of trials digging at each odor during the discrimination session. (I) Number of trials taken to reach the performance criterion in the reversal session. (J) Number of trials digging at each odor during the reversal session. *p < 0.05, **p < 0.01, ***p < 0.001. *n* = number of mice. Scale bars: 500 μm (A, F), 20 μm (B). (For interpretation of the references to color in this figure legend, the reader is referred to the Web version of this article.)

The rodent dmPFC has complex functions ranging from decision-making to action planning (Barthas and Kwan, 2017). To determine its involvement in the 4-choice test, we sacrificed control mice (4–6 weeks old) 1 h after the test and immunostained for the immediate-early gene c-Fos, the expression of which is considered a good proxy of recent neuronal activation (Kovacs, 2008). We found that the density of c-Fos+ neurons in dmPFC was significantly elevated in mice that performed the test compared to those that did not (Fig. 2A and B). Specifically, the density more than doubled in cortical layer 2/3 (*p* < 1 × 10^−4^, unpaired *t*-test; Fig. 2C), and tripled in layer 5/6 (*p* < 1 × 10^−4^, unpaired *t*-test; Fig. 2D). In contrast, the density of c-Fos+ neurons in the neighboring primary motor cortex (M1) did not change significantly after the 4-choice test (Fig. S1). Moreover, 7d RS significantly decreased the density of c-Fos+ neurons in dmPFC at the baseline (*p* < 0.05 for both L2/3 and L5/6, Mann-Whitney test; Fig. S2). These results corroborate the idea that stress affects neuronal activities in dmPFC.

To further validate the necessity of dmPFC in the 4-choice test, we silenced dmPFC neurons using the Designer Receptors Exclusively Activated by Designer Drugs (DREADDs). We injected an adeno-associated virus (AAV) encoding the inhibitory mutant human muscarinic receptor Gi (AAV8-CaMKlla-hM4D-mCherry, “DREADD virus”) into dmPFC at postnatal (P) day 21 (Fig. S3A-B). After 2–3 weeks of incubation, we injected either clozapine-N-oxide (CNO, a synthetic hM4D agonist) or saline into the mice 15 min before the test (Figs. 2E and F). We found that the two groups performed comparably in the initial discrimination phase (*p* = 0.5274, unpaired *t*-test; Fig. 2G), with similar numbers of digging at each odor (Fig. 2H). However, CNO-treated mice performed much more poorly in the reversal phase (*p* < 0.01, unpaired *t*-test; Fig. 2I). It took them significantly more correct trials (*p* < 0.05, Mann-Whitney test with FDR correction) to accomplish the task. They also made significantly more perseverative errors (*p* < 0.05, Mann-Whitney test with FDR correction) but comparable number of irrelevant and novel errors (*p* = 0.1778 for both, Mann-Whitney test with FDR correction; Fig. 2J). Furthermore, we showed that virus infection and CNO treatment *per se* did not alter the animal's performance (Figs. S3C–F). Together, these data suggest that dmPFC is indispensable for the performance of the 4-choice test.

### 3.3 7d RS promotes dmPFC dendritic spine elimination but not formation

**Fig. 3:**
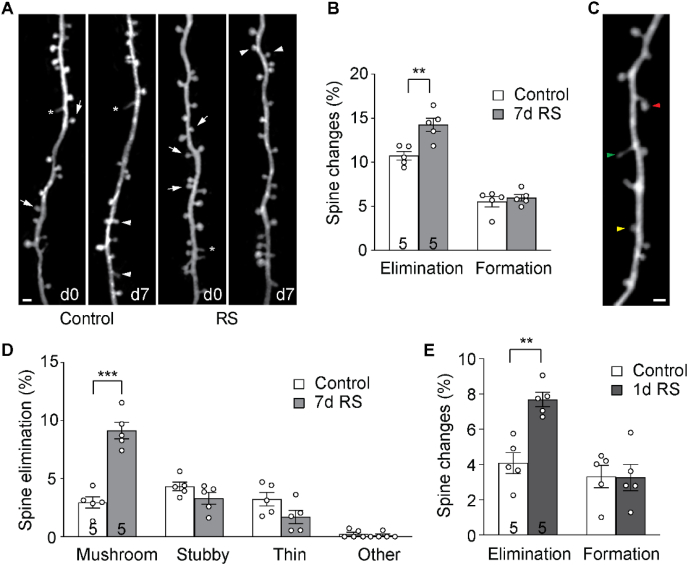
7d RS induces dendritic spine loss along apical dendrites of layer 5 pyramidal neurons in dmPFC. (A) Example of the same set of dmPFC spines imaged 7d apart in control and RS mice. Arrows: eliminated spines; arrowheads: new spines; asterisks: filopodia. Scale bar: 2 μm. (B) Percentage of spines formed and eliminated over 7d. (C) Example of different morphological categories of spines. Red: mushroom spine; green: thin spine; yellow: stubby spine. Scale bar: 2 μm. (D) Percentage of elimination of spines belonging to different morphological categories. (E) Percentage of spines formed and eliminated over 1d. *p < 0.05, **p < 0.01, ***p < 0.001. *n* = number of mice.

Dendritic spines are critical sites of information transmission between neurons (Holtmaat and Svoboda, 2009). Their emergence and disappearance reflect the reorganization of the neural circuit, which is widely believed to underlie the capacity for learning and memory (Sweatt, 2016). To examine how dmPFC spine dynamics change in response to RS, we performed transcranial *in vivo* 2P microscopy on *thy1*-YFP-H line mice, which express cytoplasmic yellow fluorescent protein (YFP) in a sparse subset of cortical layer 5 pyramidal neurons (Feng et al., 2000). We imaged segments of apical dendrites over 7 days in both control and RS mice starting around P30 (Fig. 3A). Control mice exhibited significantly higher spine elimination (10.7 ± 0.5%) than formation (5.5 ± 0.6%, *p* < 0.01, paired *t*-test). RS significantly elevated spine elimination (14.2 ± 0.8%, *p* < 0.01, unpaired *t*-test) without affecting spine formation (6.0 ± 0.4%, *p* = 0.5439, unpaired *t*-test; Fig. 3B). Classifying spines based on their morphology (Fig. 3C), we found that in control mice a comparable amount of mushroom, stubby, and thin spines were eliminated over 7 days (*p* = 0.2596, repeated measures one-way ANOVA). RS significantly elevated the elimination of mushroom spines (*p* < 0.001, unpaired *t*-test with FDR correction; Fig. 3D), but the elimination of spines in the other two categories was not changed (stubby: *p* = 0.1509; thin: *p* = 0.1499; unpaired *t*-test with FDR correction for both). Furthermore, we found that even a single session of 2 h RS could increase spine elimination significantly (*p* < 0.01, unpaired *t*-test; Fig. 3E).

### 3.4 7d RS elevates the dynamism of microglial processes in dmPFC

**Fig. 4:**
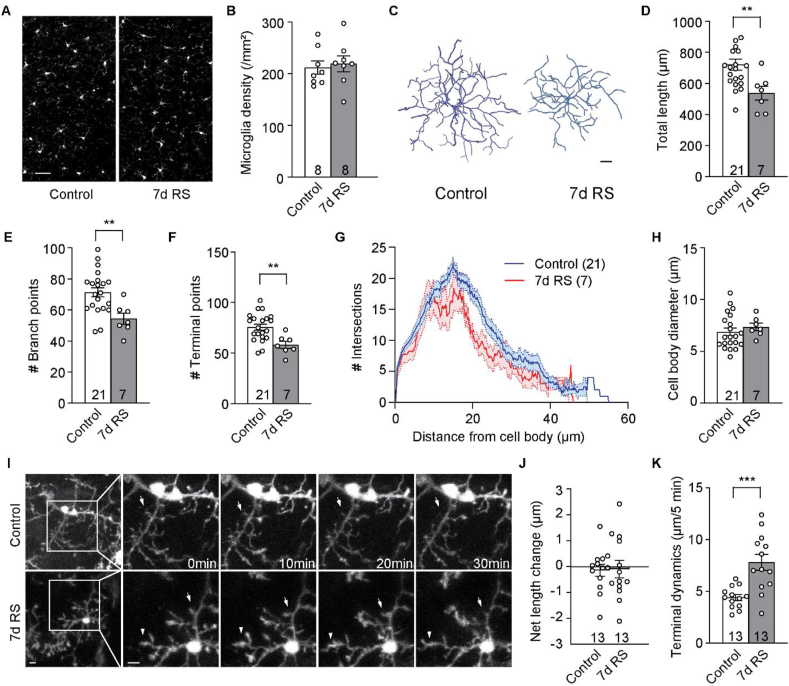
7d RS decreases the ramification and increases the process dynamics of microglia in dmPFC. (A) Representative images of Iba1 immunohistochemistry in dmPFC of control and RS mice. (B) Microglia density in dmPFC of control and RS mice. (C) Representative three-dimensional reconstructions of microglia from control and RS mice. (D–F) Analysis of total process length (D), the number of branching points (E), the number of terminal points (F) of dmPFC microglia in control and RS mice. (G) Sholl analysis of microglial processes in control and RS mice. (H) Microglial soma size in control and RS mice. (I) Representative time-lapse *in vivo* 2P images of microglia from control and RS mice. Arrows and arrowheads indicate dynamic changes. (J) Net length change of microglial terminal processes over 30 min. (K) Average terminal dynamics of microglia processes over 5 min **p < 0.01, ***p < 0.001. *n* = numbers of mice (B) or cells analyzed (D-H, J, K). Scale bars: 20 μm (A), 5 μm (C, I).

As microglia play an important role in synaptic pruning (Paolicelli et al., 2011; Schafer et al., 2012), they may be implicated in RS-induced alterations in dendritic spine dynamics. We thus compared the density, morphology, and dynamics of dmPFC microglia in RS mice and controls (4–6 weeks old). Immunohistochemistry against the microglial marker Iba1 (Imai et al., 1996) revealed comparable microglia density in 7d RS mice and controls (*p* = 0.6989, unpaired *t*-test; Fig. 4A and B). To examine microglial morphology, we used Cx3cr1-GFP mice, which selectively express cytoplasmic green fluorescent protein (GFP) in microglia (Jung et al., 2000). We imaged individual microglia in their entirety *in vivo* using transcranial 2P microscopy, traced out microglial processes, and reconstructed their morphology (Fig. 4C). The total length of microglial processes was less in RS mice than in controls (*p* < 0.01, unpaired *t*-test; Fig. 4D). The number of branching points per cell (*p* < 0.01, unpaired *t*-test; Fig. 4E) and the number of terminal points per cell (*p* < 0.01, unpaired *t*-test; Fig. 4F) were also decreased in RS mice. Sholl analysis further showed decreased microglial process complexity in RS mice (*p* < 0.05, main effect of treatment, mixed-effects ANOVA; Fig. 4G). However, there was no significant change in soma diameter (*p* = 0.4887, unpaired *t*-test; Fig. 4H). Finally, we examined the dynamism of microglial processes in anesthetized mice with 2P microscopy. We took 3D image stacks of the same microglia every 5 min over the course of 30 min. We found that the somata and main branches of microglial processes were stable throughout the imaging session, but terminal processes were quite dynamic, undergoing rapid extension and retraction (Fig. 4I). There was no net change in terminal process length over 30 min in either RS (*p* = 0.5535, one-sample *t*-test) or control mice (*p* = 0.7873, one-sample *t*-test; Fig. 4J). However, RS enhanced the dynamism of terminal processes (*p* < 0.001, unpaired *t*-test; Fig. 4K).

### 3.5 7d RS increases the contact between microglial processes and dendrites in dmPFC

**Fig. 5:**
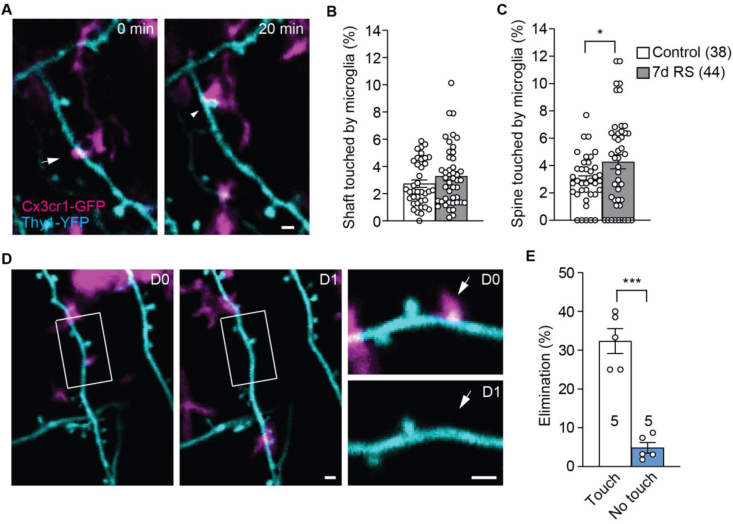
Microglia contact makes dendritic spines more prone to elimination. (A) Co-imaging dendritic spines (cyan) and microglial processes (magenta) over time shows the dynamic contact of microglia onto dendrites and spines. The arrow and the arrowhead point at dendritic shaft and spine with microglial contacts, respectively. (B) Percentage of dendritic segment length contacted by microglial processes. (C) Percentage of spines contacted by microglia processes. (D) Example of co-imaging the same dendrite and contacting microglia over 1d, showing the elimination of the spine contacted by microglia (arrow in insets). (E) Percentage of spines with and without microglial contact eliminated over 1d. *p < 0.05, ***p < 0.001. *n* = number of imaging regions analyzed (B, C) or mice (E). Scale bars: 1 μm. (For interpretation of the references to color in this figure legend, the reader is referred to the Web version of this article.)

To further examine how stress affects the interaction between microglial processes and dendritic structures, we crossed *thy1*-YFP-H mice with Cx3cr1-GFP mice and performed dual-color *in vivo* 2P imaging (4–6 weeks old). By dual-channel imaging of YFP and GFP signals and leveraging morphological features we were able to distinguish dendritic and microglial structures (Fig. 5A). We found that in both control and RS mice ~3% of dendritic shaft was contacted by microglial processes (*p* = 0.3727, Mann-Whitney test; Fig. 5B). However, RS increased the percentage of dendritic spines with microglial contact (*p* < 0.05, unpaired *t*-test; Fig. 5C). Following the fate of spines with and without microglial contact for 24 h (Fig. 5D), we found that spines with microglial contact were significantly more prone to elimination than those without (*p* < 0.001, paired *t*-test; Fig. 5E).

## 4 Discussion

The rodent PFC consists of multiple sub-regions with distinct connectivity and functions (Barbas, 2015; Barbas and Zikopoulos, 2007; Holmes and Wellman, 2009). Among them, the prelimbic and infralimbic areas of the mPFC, which is the analog of human dorsolateral PFC, have been extensively studied in the context of stress (Arnsten, 2009; Girotti et al., 2017; Holmes and Wellman, 2009; McEwen and Morrison, 2013). However, much less is known about the functional role and stress response of the dorsomedial PFC (Barthas and Kwan, 2017). We showed that dmPFC is activated during the 4-choice task, and that bilateral pharmacogenetic inactivation of dmPFC increases perseverative errors in the reversal phase, consistent with earlier lesion studies in rodents (Johnson and Wilbrecht, 2011). Furthermore, we found that adolescent mice subjected to 7d RS exhibited normal ability to learn the initial odor-reward contingency, but, when such contingency was changed in the reversal phase, they repeatedly returned to the previously correct choice, a phenomenon that is reminiscent of dmPFC inactivation and echoes human studies demonstrating that stress enhances rigid habitual behaviors at the cost of flexible behaviors (Schwabe and Wolf, 2009; Seehagen et al., 2015).

Leveraging the unique YFP expression pattern of the *thy1*-YFP-H line, we found that 7d RS accelerates the loss of cortical L5 pyramidal neuron dendritic spines, particularly mushroom spines. This parallels earlier Golgi staining studies showing that chronic RS leads to dendritic retraction and decreases spine density in the anterior cingulate cortex and the prelimbic area of mPFC, a phenotype largely consistent between L2/3 and L5 neurons (Goldwater et al., 2009; Hains et al., 2009; Li et al., 2011; Liu and Aghajanian, 2008; Radley et al., 2006, 2008). It also aligns with recent *in vivo* imaging studies in the mPFC and the sensory cortex (Chen et al., 2017, Chen et al., 2018; Moda-Sava et al., 2019). Given the diverse inputs the dmPFC receives and the distinct microcircuitry of L2/3 and L5 neurons, future studies are needed to compare the spine dynamics of L2/3 vs. L5 neurons in response to RS, and to elucidate whether RS-induced synaptic connection loss is input-specific or pervasive. The elevated loss specifically of mushroom spines is intriguing. In fact, it appears paradoxical, as large spines tend to have large synapses and are thus more stable (Holtmaat et al., 2005; Trachtenberg et al., 2002). However, this observation is consistent with previous reports of stress-induced reduction in the proportion of large spines (mushroom or stubby) in rat and mouse mPFC (Leem et al., 2020; Radley et al., 2008). It has been proposed (Kasai et al., 2003) that large spines are putative “memory spines” for long-term retention of information, whereas small spines, being more labile, are “learning spines”. The rationale is that large mushroom spines may be close to the upper limit of synaptic strength, with little room for further strengthening, and thin spines have smaller synapses and thus more potential for strengthening (Berry and Nedivi, 2017; Bourne and Harris, 2007; Hayashi and Majewska, 2005). If so, the elevated loss of mushroom spines suggests a disruption of existing memory by stress. The underlying biochemical and circuit mechanisms for such selective spine elimination remain to be elucidated.

The role of microglia in brain health and repair is increasingly drawing attention from researchers. Under the baseline condition, microglial processes constantly survey their local environment (Davalos et al., 2005; Nimmerjahn et al., 2005) and respond to neuronal activity and neurotransmission (Abiega et al., 2016; Eyo et al., 2015; Li et al., 2012). During postnatal development, microglia facilitate synaptic pruning by complement activation and phagocytosis (Ma et al., 2020; Paolicelli et al., 2011; Schafer et al., 2012); in the mature brain they regulate experience-dependent synaptic plasticity (Parkhurst et al., 2013; Rogers et al., 2011; Sipe et al., 2016; Tremblay et al., 2010). Furthermore, microglial contact may influence spine formation and stability. In the somatosensory cortex at P10, microglial contact of layer 2/3 pyramidal neuron dendrites induces local calcium transients and filopodia formation (Miyamoto et al., 2016). In the adolescent visual cortex, microglial processes localize to the vicinity of small growing spines, which are then typically lost over 2 days (Tremblay et al., 2010). In the ischemic brain, prolonged microglial contact may lead to spine loss (Wake et al., 2009). By co-imaging microglia and dendritic spines, we found that microglial processes in RS brains were more dynamic than those in control brains, with increased synaptic contact. Although only <5% of spines were contacted by microglial processes at a time, these spines were found to be more prone to elimination over 1 day compared to those without microglial contact. These results suggest a contribution of microglia to stress-induced synaptic loss. One caveat of such *in vivo* two-photon imaging studies, however, is that the axial resolution of two-photon microscopy is limited due to diffraction and aberrations induced by refractive index inhomogeneity in the living brain, which makes it insufficient to establish unambiguously the physical contact between microglial processes and dendritic structures; ascertainment of such contacts requires electron microscopy or *in vivo* super-resolution microscopy. In addition, previous studies suggest that excessive dendritic spine loss may be rescued by blocking microglia activation. For example, in mice subjected to chronic unpredictable stress, administration of diazepam or the glucocorticoid receptor antagonist RU486 limits microglial engulfment of neuronal elements and blocks stress-induced dendritic spine loss (Bollinger et al., 2020; Horchar and Wohleb, 2019). Similarly, depleting microglia or reducing microglia activation by the anti-inflammatory drug acetaminophen rescues the decreased spine density and hippocampus-dependent cognitive deficits in a mouse model of Down syndrome (Pinto et al., 2020). In an Alzheimer's disease mouse model, elimination of activated microglia also rescues dendritic spine loss and improves contextual memory (Spangenberg et al., 2016). It is unclear whether microglia actively remove synapses, or just clean up the debris after synapses have been dismantled. In either scenario, what marks specific spines for removal and what molecular signals instruct the microglia to act require further studies.

## CRediT authorship contribution statement

**Taohui Liu:** Conceptualization, Methodology, Investigation, Formal analysis, Writing – original draft. **Ju Lu:** Conceptualization, Methodology, Formal analysis, Writing – review & editing. **Kacper Lukasiewicz:** Methodology, Investigation, Formal analysis, Writing – review & editing. **Bingxing Pan:** Writing – review & editing. **Yi Zuo:** Conceptualization, Supervision, Writing – review & editing.

## Declaration of competing interest

The authors declare no competing interest.
