# Supplementary material for: Stress induces microglia-associated synaptic circuit alterations in the dorsomedial prefrontal cortex
Source: Neurobiol Stress. 2021 May 20;15:100342. doi: 10.1016/j.ynstr.2021.100342 (PMC8182072; doi:10.1016/j.ynstr.2021.100342)
Supplement: Multimedia component 1 [file mmc1.docx]

**
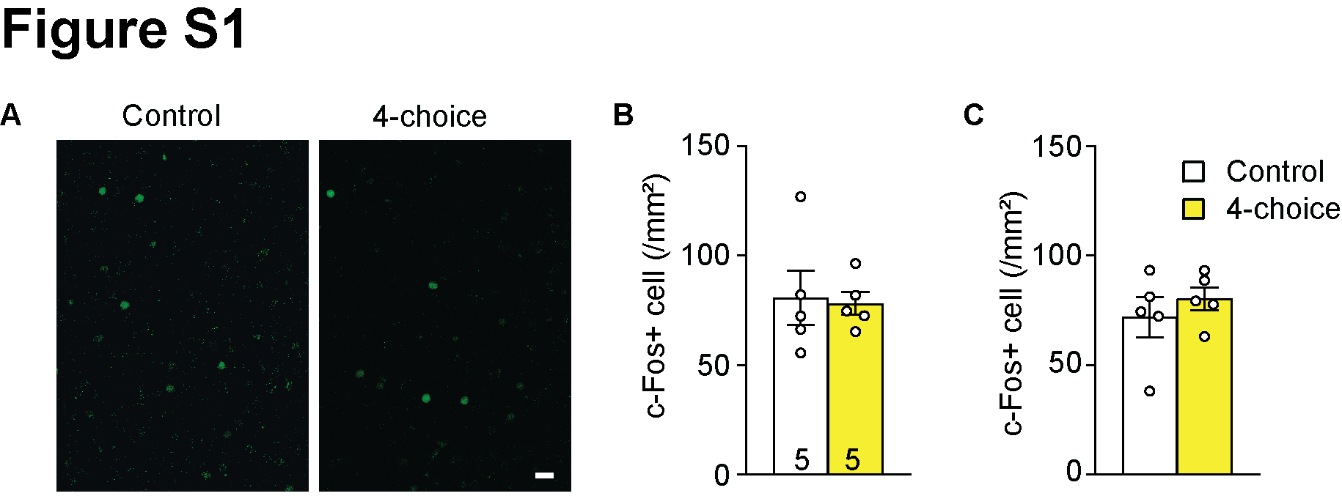
**

**Fig S1: c-Fos labeling in primary motor cortex (M1) following the 4-choice task.** (A) Immunohistochemistry of c-Fos in M1. Scale bar: 10 μm. (B and C) Performing the 4-choice task did not alter the density of c-Fos+ neurons in L2/3 (B) and L5/6 (C) in M1. L2/3: *p* = 0.8580; L5/6: *p* = 0.4447; unpaired *t*-test for both. *n* = number of mice.

**
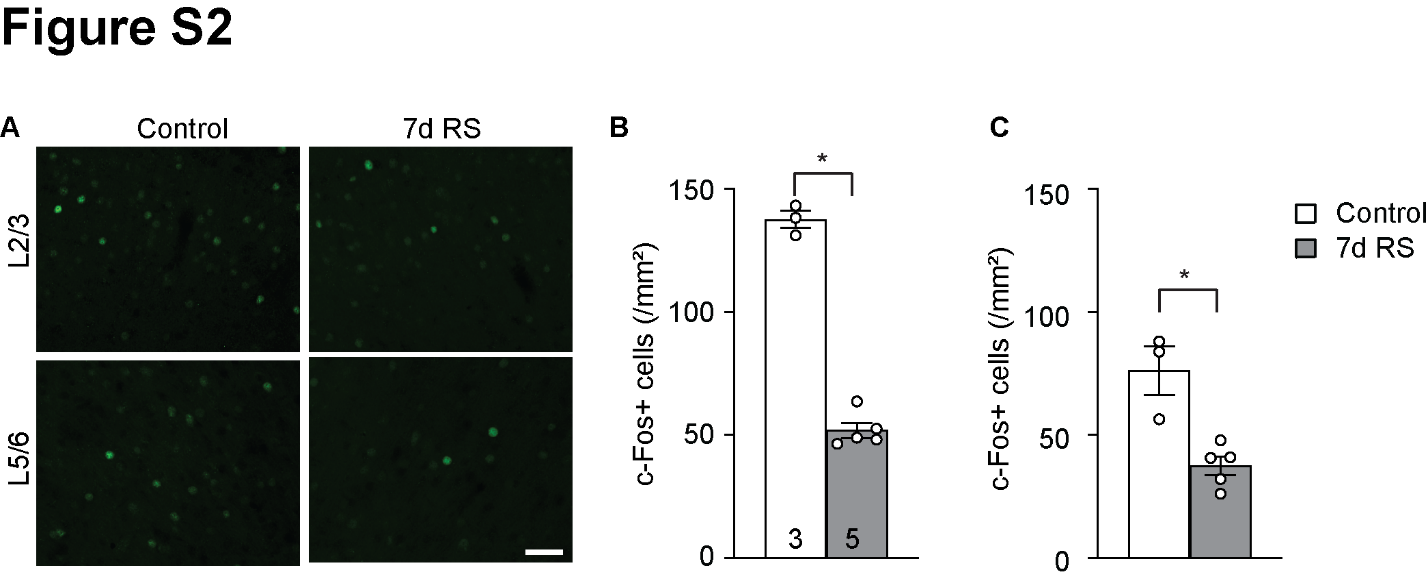
**

**Fig S2: 7d RS decreases c-Fos expression of dmPFC neurons.** (A) Examples of c-Fos immunohistochemistry in brain slices of control and 7d RS mice. Scale bar: 50 µm. (B) Density of c-Fos+ neurons in L2/3. *P* < 0.05, Mann-Whitney test. (C) Density of c-Fos+ neurons in L5/6. *P* < 0.05, Mann-Whitney test. *n* = number of mice.

**
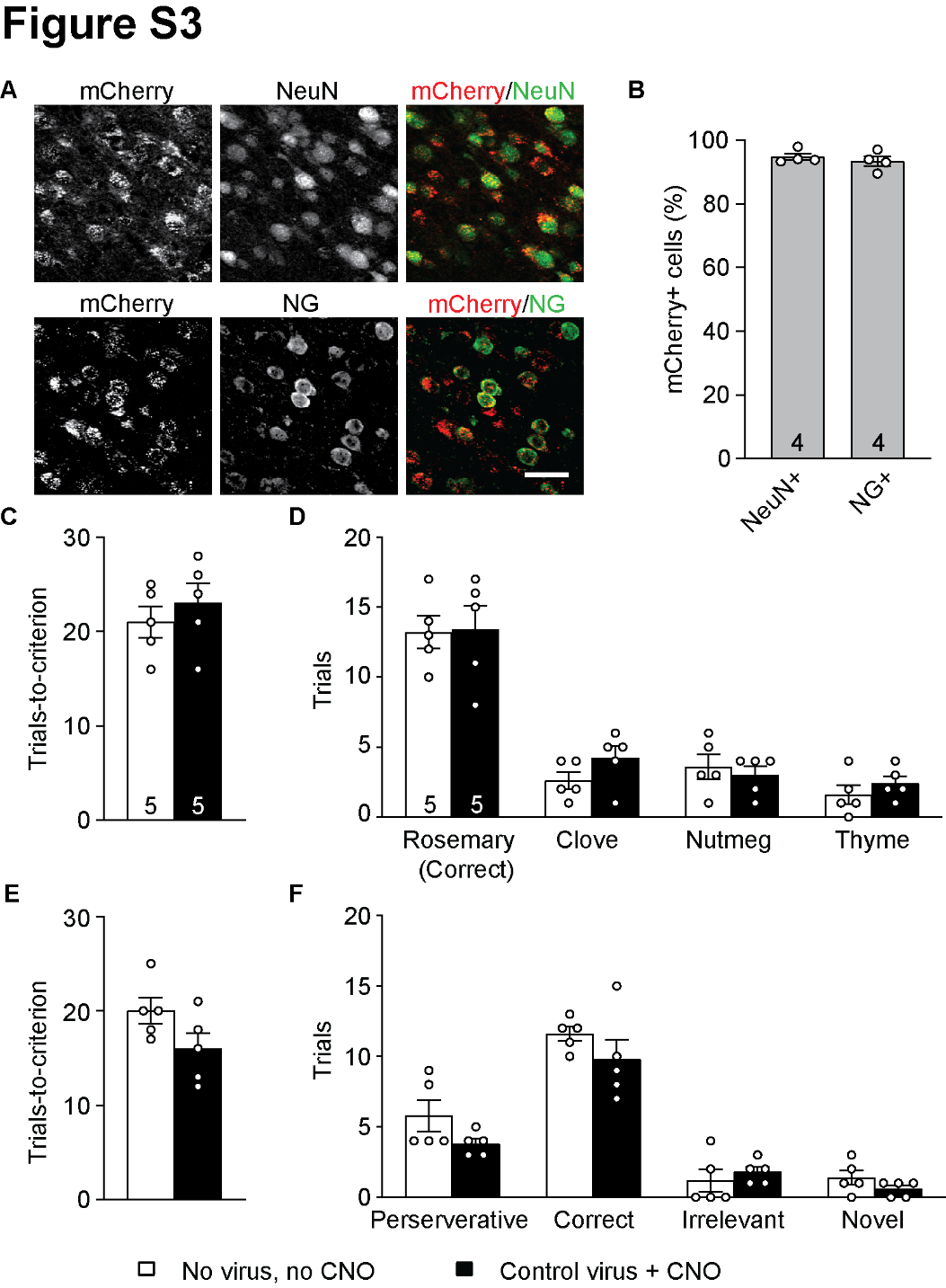
**

**Fig S3: Bilateral control virus injection into dmPFC plus CNO treatment does not alter the performance in the 4-choice task.** (A) Examples of co-labeling between the pan-neuronal marker NeuN or the excitatory neuron marker neurogranin (NG) and mCherry (co-expressed with DREADD). Scale bar: 25 µm. (B) Percentage of NeuN+ or NG+ cells that are also mCherry+. (C) Number of trials taken to reach the performance criterion in the discrimination session. Unpaired *t*-test *p* = 0.4744. (D) Number of trials digging at each odor during the discrimination session. *P* = 0.9339, 0.6692, 0.7982, and 0.7542 for rosemary, clove, nutmeg, and thyme, respectively, unpaired *t*-tests with FDR correction. (E) Number of trials taken to reach the performance criterion in the reversal session. Unpaired *t*-test *p* = 0.0992. (F) Number of trials digging at each odor during the reversal session. *P* = 0.3495, 0.3495, 0.5212, and 0.3495 for perseverative (rosemary), correct (clove), irrelevant (nutmeg), and novel (cinnamon), respectively, unpaired *t*-tests with FDR correction. *n* = number of mice.
